# Data management in diabetes clinical trials: a qualitative study

**DOI:** 10.1186/s13063-022-06110-5

**Published:** 2022-03-03

**Authors:** Aynaz Nourani, Haleh Ayatollahi, Masoud Solaymani Dodaran

**Affiliations:** 1 Department of Health Information Technology, Urmia University of Medical Sciences, Urmia, Iran; 2 Health Management and Economics Research Center, Health Management Research Institute, Iran University of Medical Sciences, Tehran, Iran; 3 Department of Health Information Management, School of Health Management and Information Sciences, Iran University of Medical Sciences, Tehran, Iran; 4 Department of Epidemiology, Iran University of Medical Sciences, Tehran, Iran

**Keywords:** Clinical trial, Diabetes, Data management, Clinical data management, Medical informatics

## Abstract

**Background:**

Clinical trials play an important role in expanding the knowledge of diabetes prevention, diagnosis, and treatment, and data management is one of the main issues in clinical trials. Lack of appropriate planning for data management in clinical trials may negatively influence achieving the desired results. The aim of this study was to explore data management processes in diabetes clinical trials in three research institutes in Iran.

**Method:**

This was a qualitative study conducted in 2019. In this study, data were collected through in-depth semi-structured interviews with 16 researchers in three endocrinology and metabolism research institutes. To analyze data, the method of thematic analysis was used.

**Results:**

The five themes that emerged from data analysis included (1) clinical trial data collection, (2) technologies used in data management, (3) data security and confidentiality management, (4) data quality management, and (5) data management standards. In general, the findings indicated that no clear and standard process was used for data management in diabetes clinical trials, and each research center executed its own methods and processes.

**Conclusion:**

According to the results, the common methods of data management in diabetes clinical trials included a set of paper-based processes. It seems that using information technology can help facilitate data management processes in a variety of clinical trials, including diabetes clinical trials.

## Introduction

To create a better future for patients with diabetes and to reduce health care costs, conducting research is the first and the most important step [1]. Many universities and research centers, such as the Juvenile Diabetes Research Foundation (JDRF) and National Institute of Diabetes and Digestive and Kidney Diseases (NIDDK), believe that research about diabetes is a global task to identify new treatment approaches, improve current medications and diagnostic methods, and make the life more pleasant for patients with diabetes [2]. There are various types of diabetes studies [3] and one of the most significant ones is conducting clinical trials in which safety and efficacy of novel types of interventions are assessed on humans for prevention and treatment of the disease [4].

Regardless of the complexity of clinical trials, one of the crucial components that act as a bridge between the research idea conceptualization and dissemination of the final results is the clinical data management (CDM). Clinical data management includes all data management activities in a clinical trial project and is an influencing factor in the success or failure of a clinical trial. For instance, a clinical trial with an appropriate scientific design, but incoherent and weak data management method can end up with poor quality data that is not worth analyzing and citing [5].

Clinical data management is defined as settings and policies required to collect, control, protect, present, and enhance the value of data and information assets in the field of clinical trials [6]. It is an important activity in clinical trials that leads to generating high-quality and reliable data [7]. The most important tasks in the clinical data management process are designing case report forms (CRFs), annotating CRFs, creating databases, entering data, validating data, managing inconsistencies and resolving data disputes, medical coding, data extraction, database locking, documenting CDM process, and providing data security during a clinical trial [4, 6, 8–11]. This process, especially in multicenter clinical trials, in which a large number of participants and researchers are involved, is so complicated and cannot be performed manually [12]. Therefore, electronic clinical data management has been suggested to facilitate the whole process [13, 14].

Electronic clinical data management requires adequate hardware, software, and communication technologies as well as settings, data collection policies, and controlling data quality and security [15, 16]. Although many strategies and interventions have been proposed to reduce mistakes in the process of clinical data collection and management, it seems that there are still many other factors that need to be taken into account. Some strategies have only focused on developing and using standards, training the staff, monitoring the data, and designing intelligent case report forms [16], and numerous studies have addressed the strengths and weaknesses of research centers in conducting data management and their compliance with clinical trial standards [15, 17, 18].

As the main requirement for designing and implementing an electronic clinical data management system is investigating the clinical data management processes in a clinical trial, the present study aimed to explore data management processes in diabetes clinical trials in three research institutes in Iran.

## Methods

The present study was conducted qualitatively in 2019. The settings of the study were three endocrinology and metabolism research institutes affiliated with three medical universities in Iran. They were also responsible for conducting clinical trials related to endocrine and metabolic disorders including diabetes. Prior to the research, ethical approval was obtained from the ethics committee of the Iran University of Medical Sciences (IR.IUMS.REC 1395.9321481003).

In this study, purposive sampling with maximum variation was used, and various groups of researchers in terms of age, sex, specialty, work experience, and conducting diabetes clinical trials were invited to participate in the study. The total number of eligible participants was 25. Some of the criteria for selecting the participants were willing to participate in the interview, having at least five years of work experience, and conducting at least four diabetes clinical trials as the main investigator.

To conduct the interviews, one of the researchers (AN) attended the interviewee’s workplace with prior time arrangement. An interview guide was used for asking questions. It was prepared based on a literature review [7, 9, 11, 19–29] and contained 14 questions about different types of diabetes clinical trials, data required for diabetes clinical trials, data entry methods, data management tools, data quality and security management methods, reporting methods, and data management standards. To collect data, the interviews were recorded, and notes were taken when needed. In some cases, where the researcher was not allowed to record the voice of the interviewee, the content was written. Before conducting the interviews, the interviewees were asked to sign an informed consent form and complete demographic questions. Interviews continued until data saturation was reached.

All interviews were transcribed, coded, and analyzed using the thematic analysis method, and MAXQDA software was used to facilitate coding data. Finally, the themes, subthemes, and categories were extracted and reported narratively. A summary of the results was also given to the participants to check the accuracy and confirm the credibility of the results.

## Results

In this study, 16 out of 25 eligible participants took part in the interviews. The average time of the interviews was 32 min. The demographic characteristics of the participants are presented in Table 1.

**Table 1:** Characteristics of the participants

Variable		Frequency (%)
Sex	Male	5 (31.3%)
	Female	11 (68.7%)
Age	36–40	4 (25.0%)
	41–45	1 (6.3%)
	46–50	4 (25.0%)
	51–55	7 (43.7%)
Specialty	Endocrinology	6 (37.3%)
	Nutrition	3 (18.7%)
	Epidemiology	2 (12.5%)
	Obstetrics and gynecology	1 (6.3%)
	Pharmacology	1 (6.3%)
	Health education	1 (6.3%)
	Molecular medicine	1 (6.3%)
	Clinical pathology	1 (6.3%)
Work experience (year)	6–9	5 (31.3%)
	10–13	8 (50.0%)
	14–17	3 (18.7%)
Number of completed diabetes clinical trials	4–6	12 (75.0%)
	7–9	2 (12.5%)
	10–12	2 (12.5%)

As Table 1 shows, the majority of the participants (*n*=11, 68.7%) were female. Also, half of the interviewees (*n*=8, 50.0%) had a work experience between 10 and 13 years and most of them (*n*=12, 75.0%) had an experience of conducting at least 4–6 diabetes clinical trials. The five main themes that emerged from the qualitative study are summarized in Table 2.

**Table 2:** Themes, subthemes, categories

Theme	Subtheme	Category
Clinical trial data collection	Data collection methods	Electronic records
		Paper-based records
	Data entry responsibility	Researchers
		Data entry operators
Technologies used in data management	Hardware	Personal computer
	Software	Statistical software
		Data management software
	Telecommunication technologies	Intranet
		Internet
Data security and confidentiality management	Managerial dimension	Physical safeguards
		Closed-circuit television camera
		Data de-identification
	Physical dimension	Using safe file cabinets
		Locking rooms
	Technical dimension	Using username and password
		Using antivirus software
		Data backup
		Role-based access control
Data quality management	Quality assessment during data collection	Accuracy
		Precision
		Timeliness
		Completeness
	Quality assessment during data entry	Consistency
		Relevancy
Data management standards	Data collection, storage, and exchange standards	Good Clinical Practice (GCP)
	Data coding and adverse event reporting standards	Food and Drug Administration (FDA) standard

### Theme 1: clinical trial data collection

The findings of the current study revealed that diabetes clinical trials data were collected either electronically or manually using paper-based records. The data collection responsibility was mainly assigned to the researchers and in a few cases, data entry operators were asked to collect the data. However, most of the interviewees stated that in their research institute, diabetes clinical trials data were mainly collected manually. For example, one of the interviewees mentioned: “In the clinical trials I have conducted so far, I had a paper-based questionnaire for each patient and recorded all the necessary data in the questionnaire. The contracting laboratory also sent us some information in the form of paper-based reports, and we rewrote these reports into the corresponding fields in the questionnaire. It was all performed manually.” A small number of the researchers who previously collaborated in multicenter clinical trials stated that they collected the data electronically using web-based software provided by a pharmaceutical company.

The interviewees believed that data entry responsibility was one of the most important roles in clinical trials, and researchers themselves or data entry operators should be in charge of entering data. Most of the researchers indicated that they, as the principal investigators, were responsible for entering data. Some interviewees mentioned that they asked a data entry operator to help them. A data entry operator was a skilled person in computer science, biostatistics, or other related specialties and responsible for entering a large amount of data into the computer or paper-based records. In this regard, one of the researchers said: “Researchers do not usually enter data as it is time consuming. We definitely hire a data entry operator to collect and enter data. This person accompanies us. For instance, I ask the patient some questions, and the data entry operator enters the data in the questionnaire and finally enters them into the final EXCEL file.”

### Theme 2: technologies used in data management

Regarding the technologies required for data management in clinical trials, most of the interviewees stated that they must have access to sufficient hardware, software, and telecommunication technologies to manage the data effectively. Although hardware availability is essential for running clinical data management software and data exchange, most researchers pointed out that they needed more advanced equipment in their research institutes to be able to manage clinical data effectively. For example, one of the interviewees noted: “At this center, only a medium-speed personal computer is provided for each researcher. We do not have tablets, laptops, and other mobile hardware to take them to the patient’s bedside and enter the data at the point of care. In this respect, our center is weak.”

Regarding software, it can be mentioned that data management and analysis software (statistical software) is one of the most important technologies used in clinical trials. All interviewees stated that they took advantage of statistical software, especially EXCEL and SPSS, to record, manage, and analyze their study data. Moreover, some researchers noted that they had some experience of using clinical data management software in other settings. For instance, one of the endocrinologists said: “I used web-based data management software in a clinical trial sponsored by a pharmaceutical company; however, I did not do the data management work myself using that software. The company only allowed us to enter and view the data.”

In addition, as the researchers required sharing study data with colleagues, research assistants, trial sponsors, and other related organizations, the use of telecommunication technologies was essential in the research centers, especially those in which multicenter clinical trials were conducted. In this study, telecommunication technologies employed in the research centers were divided into two categories of the Internet and Intranet. One of the researchers noted: “We use an internal network, i.e. Intranet to communicate with our colleagues. This is very good because, for example, I do not have to move the data to the flash [drive]. The lab also sends the lab data file to my system. We also use the Internet for cases where the Intranet is not responsive.”

### Theme 3: data security and confidentiality management

One of the issues addressed by the interviewees was related to the security and confidentiality management of diabetes clinical trial data. This theme included managerial, physical, and technical dimensions.

In terms of managerial dimension, all research institutes used physical safeguards and closed-circuit television camera (CCTV). In addition, the researchers noted that they used encryption methods and de-identified participants’ data to maintain confidentiality issues. In this regard, one of the researchers stated: “I assign a code to the patient from the very beginning when I enter them into my study, and then I use it on their blood samples, on the patient record, and most importantly on the final file that needs to be analyzed.”

In terms of physical security, since most of the clinical trial data were collected in paper-based records, most researchers believed that data security management is merely providing physical security for this type of data. They used safe file cabinets for storing case report forms and locked the rooms with the keys that were only available to the principal investigator. However, these cabinets were not fire and water-resistant. Furthermore, the buildings and the rooms were only equipped with fire alarms and fire extinguishers.

Some researchers discussed the technical solutions to secure electronic data. These solutions included using username and password, antivirus software, data backup, and role-based access control. In this respect, one of the researchers expressed that: “The researchers themselves should be responsible for maintaining the security of their data. For example, we installed antivirus on our systems. We all have usernames and passwords, and sometime, we back up the data, especially when we have to prepare the mid-term and final reports.”

Furthermore, as some clinical trials were conducted using the financial support of private companies and organizations, it was necessary to send the data and their final reports to the research sponsor. In this regard, one of the interviewees stated: “The parties to the clinical trial contract, which may be research centers, pharmaceutical companies, and other support companies, have the right to access the data and final reports of clinical trials, and it makes sense to have at least one copy of them*.*”

### Theme 4: data quality management

Data quality management was another theme extracted from the research findings and was divided into the quality assessment during data collection and data entry.

Given that a large part of the data quality assessment process is performed during data collection, most of the interviewees stated that they avoided collecting irrelevant data and minimized missing data by spending enough time and being precise during data collection. In general, the interviewees implied that it would be more beneficial to collect the right, complete and accurate data at the right time to maintain the validity of data. So that there will be no need to spend a lot of money and time to re-check the quality and validity of data in the future. As one of the researchers noted: “It is better to get everything planned from the very beginning. I mean, we should have a plan specifying what data we must collect at the time of intervention and patient visit, and should pay adequate attention to what scales we employ to collect the data. By doing so, a lot of data are collected and we do not have the problem of incomplete or inaccurate data.”

There was always a possibility of making mistakes during reading data from paper-based records and entering them into the computer which might reduce the quality and validity of the clinical trial data. The interviewees pointed out that at this stage, the compatibility of the entered data with other data and the relevancy of the data should be checked in order to report a high-quality report. In this regard, one of the researchers said: “I prefer to check the data in several steps. The first step is to re-examine every piece of data and to check if it is entered correctly. Another step is to request an expert to prepare the EXCEL and SPSS files for me. For example, if the wrong data are entered, it will not be accepted. A range should be set for the data so that a message will be sent if it is out of the range. A rule should be defined for a series of fields. For example, if the type of diabetes is gestational diabetes, the user cannot enter a number higher than 9 months in the disease duration field.”

### Theme 5. data management standards

Another theme was data management standards in clinical trials which were divided into two subthemes as follows: (1) data collection, storage, and exchange standards and (2) data coding and adverse event reporting standards. The Good Clinical Practice (GCP) standard is one of the important standards that show how systematically collects, stores, and exchanges clinical trial data. Although most of the interviewees stated that they attended GCP standard training courses, it seemed that sometimes this standard was not comprehensively used in relation to the clinical trial data. According to the interviewees, the researchers had difficulties with clinical trial data management and the use of information technology for managing these data.

Coding clinical trials data and coding and reporting adverse events (AEs) that may occur for patients during interventions both are two main aspects of clinical data management. Although all interviewees expressed the importance of using these standards, they did not use them in practice, mainly because they were not trained in using them. Instead, if they experienced adverse events during a clinical trial, they documented and described them. For example, one of the researchers said: “These standards are undoubtedly beneficial; however, neither we are familiar with them nor we have been taught in this respect. So far, we have recorded the data following the traditional format. We have also used a descriptive method to report adverse events.” Another interviewee noted: “As I know, some pharmaceutical companies use Food and Drug Administration (FDA) standards for reporting adverse events; however, I have never used them personally.”

## Discussion

Clinical trials are primarily conducted to answer specific questions about the effectiveness and safety of a particular product or a treatment method. However, answering these questions depends on the proper data collection and management [4]. The results of the present study revealed that the data collection methods used in diabetes clinical trials varied in the settings of the study. It means that in most cases, data were collected using paper-based records and in a small number of studies funded by pharmaceutical and biotechnology companies, this process was completed electronically. In addition, the findings of the current study indicated that collecting paper-based data is a time-consuming activity, especially in multicenter diabetes clinical trials. For example, in a large multicenter study, the data were recorded on paper-based case report forms and sent to the Clinical Trials Coordination Center (CTCC) using the mail, which led to the waste of time, difficulty in tracking data, and compromising data security. Similarly, Gao et al. reported that paper-based case report forms make the proper conduction of clinical trials difficult [30]. In another study, Cragg et al. implied that the use of paper-based case report forms, especially in multicenter clinical trials reduces the response rate of the research centers [31]. Therefore, the use of informatics in clinical trials data management and designing and implementing new information systems are suggested to facilitate the process of data collection and improve the quality and confidentiality of data.

Due to the difficulties experienced by clinical trials researchers in relation to data management issues, recruiting data management experts has been suggested [32, 33]. Many well-known research centers have a team to collect, manage, protect, and monitor data. This team usually includes experts in clinical data management who have no intervening roles in the study, and only have to collect data correctly and monitor data quality and security. ^33^ In the present study, the findings showed that there was not adequate staff in the research institutes to collect and manage clinical trials data. In particular, data management experts had not been employed in these institutes to monitor data quality issues. In fact, in most cases, the principal investigator and their clinical trial colleagues collected the data using paper-based case report forms and in a few cases, data entry operators assisted the researchers in entering data into the statistical software. According to the results, although collecting data by the researchers might improve the quality of data, it was a time-consuming task for them. On the other hand, data entry operators could make mistakes during data entry, as they might not be familiar with clinical data. Similarly, Durkalski et al. noted that there is a shortage of experts in data management in most clinical trials, and there are daily challenges regarding the data management and quality control of trials [34]. In another study, Ohmann et al. showed that the lack of human resources specialized in clinical data management should be considered as an important challenge in the academic research centers [28]. We therefore suggest that it is important that research centers pay particular attention to recruiting data management experts who are familiar with the principles of data management and are able to monitor data quality either in paper-based or electronic-based systems.

According to the findings of the current study, in most diabetes clinical trials, the process of data entry to the statistical software was performed at the end of the study due to the shortage of staff or time constraints. Therefore, not collecting data in a timely manner could lead to reducing data quality and creating additional workload to investigate and resolve potential data quality issues. In this regard, Das et al. pointed out that data management, especially in multicenter trials, should be supported by an expert who follows the rules. In particular, in large-scale multicenter studies, data collection, storage, and transmission must be performed timely and appropriately [35]. Therefore, it seems that recruiting the data management experts and using data management systems in the settings of the study and other similar institutes can help the researchers to solve these problems effectively and reduce their workload.

Currently, information and communication technologies (ICT) are considered as an integral part of clinical trials [17, 36–38]. The results of the present study showed that all three research institutes were equipped with adequate hardware and communication infrastructure. However, instead of clinical data management software, they used statistical software to manage and analyze their study data. In this regard, Kuchinke et al. expressed that data management in clinical trials faces several problems and challenges due to the heterogeneity of software products for clinical data management as well as the complexity of computer centers and information technology infrastructure. They noted that usually there is a lack of an appropriate computer center in the academic research centers which makes managing large multicenter clinical trial data difficult [17]. Similarly, Ohmann et al. highlighted the limitations of hardware and software technologies available in the research centers [28]. In other studies, poor communication infrastructure has been addressed as a problem for clinical trials data management in developing countries [15, 18]. As conducting diabetes clinical trials is important and a large part of clinical trials is devoted to diabetes, it seems that developing a diabetes clinical data management system can be useful for the researchers to collect, store and disseminate data in a well-defined format [39, 40].

One of the significant issues in clinical trial data management is protecting the security and confidentiality of the participants’ data during the data collection, storage, and transmission processes [23]. Protecting the security and confidentiality of this type of data can be performed in various ways such as restricting access, backing up the database, and encrypting the data [15]. The results of the present study demonstrated that the security and confidentiality of data in diabetes clinical trials could be considered in managerial, physical, and technical dimensions. However, it seemed that in these three research institutes, the most routine and common security approaches were used to protect data. Obviously, by using data management systems, more complicated technical solutions can be applied and participants’ data can be protected more rigorously in the settings of the study. In the studies related to developing clinical trial data management systems, the use of usernames and passwords was considered as one of the primary layers of data security [36, 41–44]. Moreover, periodic backup of the collected data and storing data in a safe place have been emphasized in some studies [15, 36, 44]. However, it seems that these solutions are not enough to maintain data security and most data management systems use complementary methods such as role-based access control [45–47], data encryption [45, 48, 49], and event log files [15, 49]. As data security maintenance is one of the important activities in the process of clinical data management [50], we suggest that attention needs to be paid to investigate users’ requirements and include related security features in the systems designed for any research institute.

The quality of clinical trials results is strongly influenced by the quality of data [51]. Nahm et al. believed that the lack of planning for data quality management in clinical trials can lead to invalid results and thus uncertainty about the effectiveness and safety of the study [52]. The results of the present study demonstrated that data quality assessment in diabetes clinical trials was performed manually at the time of data collection and during data entry in the settings of the study. Moreover, there was no specific plan for data quality control in these research institutes, and the researchers themselves were responsible for creating a data quality control plan. Other studies have also discussed the importance of data quality management in clinical trials [51–53]. In some studies, various layers of validation and a variety of quality control tools, such as a combination of rule-based validation and range control approaches were considered in the development of clinical data management systems [36, 45, 47]. However, in some systems, very basic and common methods, such as manual checking was used to control the quality of data [15, 46, 48, 49]. Müller et al. used only a combination of range control and format control methods to prevent the entry of junk and irrelevant data [39]. It seems that data quality assessment can be improved by using data management systems in which different levels of checking and validation can be defined. For example, in addition to the quality control during data entry, quality assessment during freezing data and defining the locking of the database are important in supporting the quality of results.

Regarding clinical trial data management standards, the results of this study indicated that although the participants were familiar with some standards like GCP, there were still some weaknesses in the process of clinical trial data management mainly due to the limited use of data management systems. This issue could affect data quality and confidentiality, and compromise ethical aspects of a clinical trial. Although the researchers attempted to manage the data precisely by themselves, there might be a room for human errors, especially in relation to the multicenter clinical trials with a large volume of data. Similarly, in Kuchinke et al.’s study, the inadequacy of electronic infrastructure and the lack of clinical trial data management systems were considered among the reasons for non-compliance with the related principle of GCP standards [17]. Therefore, it is important that researchers are provided with adequate information systems to be able to follow GCP data management standards. These systems can also facilitate medical coding and reporting adverse events in a standard format rather than describing an event by a researcher.

Overall, it seems that high-quality clinical trial data management processes are necessary to improve the quality of data and to facilitate this process for the researchers. This can happen either by recruiting data management experts, designing and implementing data management systems, or training the researchers in using the standards.

## Research limitations

In the present study, various groups of researchers involved in diabetes clinical trials were interviewed. However, this study was conducted only in three endocrinology and metabolism research institutes and the number of the interviewees was limited. These research institutes might be different from many other research institutes in the country mainly in terms of the equipment, infrastructure, and routine workflows. Therefore, the results need to be examined using a bigger sample size. Moreover, other research methods can be used to validate the results derived from this study.

## Conclusion

The aim of this study was to explore data management processes in diabetes clinical trials in three research institutes in Iran. The findings of this study revealed that although data management was an integral part of a diabetes clinical trial, basic tools and a set of paper-based processes were used to meet the researchers’ requirements. In addition, the results showed that researchers usually did not use clinical trial data management software to facilitate collecting and sharing clinical trial data. Therefore, it seems that designing and implementing clinical data management software for these institutes and similar settings can help them to conduct future clinical trials in a more systematic way, which in turn helps to improve efficiency and effectiveness.

## Data Availability

All data generated or analyzed during this study are included in this article.

## Abbreviations

JDRF: Juvenile Diabetes Research Foundation
NIDDK: National Institute of Diabetes and Digestive and Kidney Diseases
CDM: Clinical data management
CRFs: Case report forms
GCP: Good Clinical Practice
AEs: Adverse events
CTCC: Clinical Trials Coordination Center
ICT: Information and Communication Technologies
